# Stem cells and art: an interview with Yi Arial Zeng on the promising future of the stem cell field

**DOI:** 10.1038/s42003-021-02169-z

**Published:** 2021-06-02

## Abstract

Yi Arial Zeng has been a Principal Investigator at the Shanghai Institute of Biochemistry and Cell Biology (SIBCB), Chinese Academy of Sciences since 2010. In this interview she talks about her research, but also shares her experience of the challenges facing a group leader and highlights the importance of art and beauty in science and beyond.

**Figure:**
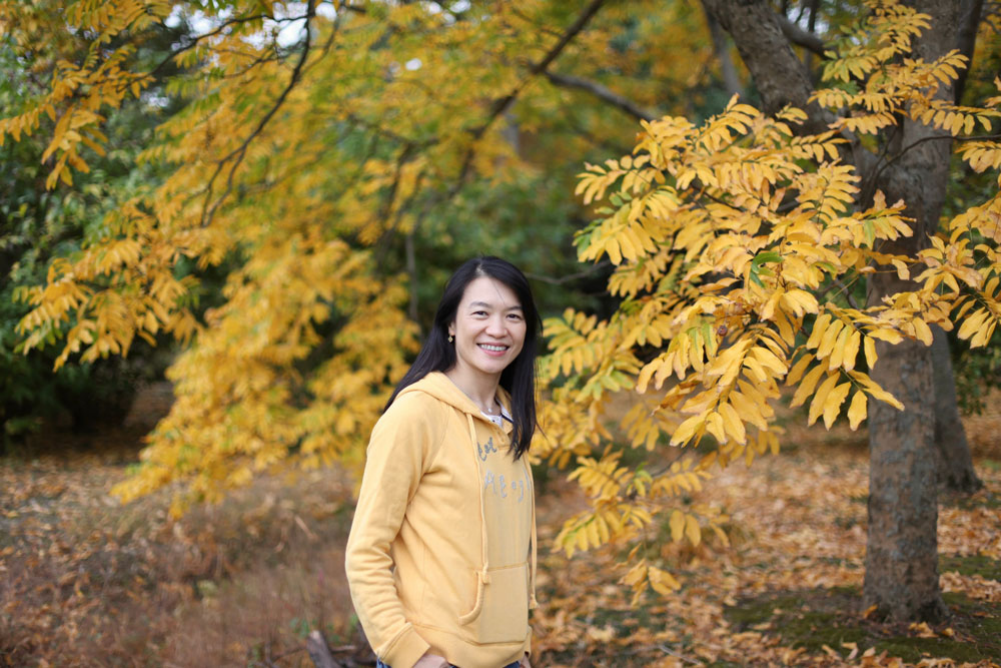
Yao Cong

Please tell us about your research interests!

My research interest is to understand the regulatory mechanisms of adult stem cells, and the interaction between stem cells and their niche. We focus our efforts on tissues that their stem cells express a surface marker Procr (protein C receptor), e.g. the mammary gland and the pancreatic islets.

Our goal is to determine how the stem cell regulatory mechanisms have deviated in diseases, and to learn how to control the players in these machineries in vivo, as well as to provide ever-expanding stem cells in vitro for the purpose of regenerative medicine.

So much has been learned about stem cell biology in the past 10 years. What are you hoping will be achieved in the stem cell field in the next 10 years?

In the embryonic stem cells and induced pluripotent stem cell field, we hope to see various types of differentiated functional cells ready for off-the-shelf cell therapies.

In the adult stem cell field, both in vitro and in vivo research looks promising. Stem cells or stem cell-derived organoids can provide a system for drug sensitivity tests, and more directly, present a cell source for regenerative medicine. In vivo, we shall understand the biology of the resident tissue stem cells and find ways to stimulate their activation to achieve regeneration in situ.

What is your favourite memory from your scientific career so far?

There are many, for example seeing my students opening champagne for the celebration of a publication, or having delivered what felt like a spectacular talk, where I connected with, and won over the audience. My recent favorite moment is a rather “small” case—an update from my student Bai. We had planned the experiment a couple of years ago and we took time and risk to build very elegant animal models. Last week, Bai showed me the first result and we were both excited yet holding back the ecstasy because it needs more biological repeats. The rest of the week, whenever I thought of the result, I was filled with joy. I reminded myself, that is the reason I stay in this job. I want to remind my lab members it is the moments like that—not the papers, not the glory—that are the reason we stay in science.

What would be your advice for women who are considering a career in scientific research?

My main advice would be: Know what you want and don’t give up! Besides, the empathy we have helps to connect with people and nurture relationships. I find it rewarding when I connect with my students and help them progress in their career. It takes a lot to run a lab, but it also brings plenty of rewards.

What is the main challenge you have faced as a PI?

The challenge is ever-changing as a PI. In my early years, I found it challenging to be a cheerleader for the entire lab while I am still struggling to transition from a postdoc to a group leader. The responsibility for everyone to feel excited about their projects, to engage, and to succeed is enormous. Stepping into the 2^nd^ decade of my job, the lab becomes more mature and is filled with smart and dedicated young people. I sometimes need to make strategic decisions to terminate projects to focus on the key directions. I find it is crucial yet challenging, considering that the desire to explore must be acknowledged too.

You have a gallery of amazing images from your research on your home page. Would you say that art is important to you as a scientist?

Fun fact, my name Yi in Chinese means art, reflecting my parents’ wish for me to be artistic. I am indeed drawn to beautiful images, once being an amateur wedding photographer during my postdoc days. After having my own lab, I started decorating my own office, which is now called by my colleagues “the most beautiful office” on campus. I am very pleased that my students also share the same good taste. They are fond of capturing the surreal beauty hidden under the microscope. Every year in May, we have a Lab Open Day for elementary and high school students. To prepare souvenirs, we collected these images to make postcards, hoping to plant a seed for the young to chase truth and beauty. We post this gallery on our home page, to recognize and applaud minds discovering science from an artistic view. So yes, I would say art is important to me as a scientist and beyond.

*This interview was conducted by Deputy Editor Christina Karlsson Rosenthal.*

